# The cooperative behavior and intention to stay of nursing personnel in healthcare management

**DOI:** 10.25122/jml-2022-0277

**Published:** 2022-10

**Authors:** Nurmiati Muchlis, Haeril Amir, Desy Dwi Cahyani, Rizqy Iftitah Alam, Nurfitri Landu, Mikawati Mikawati, Nur Febrianti, Junaidin Junaidin, Mei Rianita Elfrida Sinaga

**Affiliations:** 1 Faculty of Public Health, Universitas Muslim Indonesia, Makassar, Indonesia; 2 Midwifery Department, Poltekkes Kemenkes Malang, Malang, Indonesia; 3 Scholl of Nursing, STIK Makassar, Makassar, Indonesia; 4 Nursing Study Program, STIKES Panakukang, Makassar, Indonesia; 5 Nursing Study Program, Akademi Keperawatan Justitia, Palu, Indonesia; 6 Nursing Study Program, Stikes Amanah Makassar, Makassar, Indonesia; 7 Nursing Study Program, Sekolah Tinggi Ilmu Kesehatan Bethesda Yakkum Yogyakarta, Yogyakarta, Indonesia

**Keywords:** cooperative behavior, intention to stay, nursing personnel, turnover

## Abstract

The management of human resources is essential in a hospital, and its success can be seen based on the turnover rate of nursing personnel (nurses and midwives). In a hospital, the nursing personnel represents the largest number of professionals, and its performance greatly affects the effectiveness of services for patients. This study examined how organizations can predict turnover rates through intention to stay. Furthermore, this study aimed to explain the influence of cooperative behavior (both perceived external prestige and internal respect that affects organizational identification), which correlates with the intention to stay of nursing personnel. This quantitative research had a cross-sectional design, using a survey. The population involved non-permanent workers in five private and government hospitals. The sample consisted of 147 respondents. This study indicates that cooperative behavior showed positivity and significantly influenced the intention to stay, based on a 95% confidence degree. Perceived external prestige (p=0.009) and perceived internal respect (p=0.002) showed positivity and significantly influenced organizational identification. Perceived internal respect directly influenced the intention to stay (p=0.000), and organizational identification showed positivity and significantly influenced the intention to stay (p=0.000). Hospital management is more active in improving efforts and programs to improve the behavior of cooperatives, which is actually more dominant in non-financial aspects.

## INTRODUCTION

The management of hospital human resources is essential, and its success can be seen in hospitals that experience a turnover rate in nursing personnel that increases every year. Nursing personnel is the most demanded profession in a hospital, being very important for service effectiveness. Previous research conducted by Sinding reported that since 2008, approximately 81% of companies had considered their employees a significant priority [1]. This rate increased by 40% in 2007. Employee turnover becomes a betrayal since it harms the company. The turnover rate is calculated based on the amount of voluntary exit for a given period divided by the average number of existing employees for a given period (the number of beginning years and the end of the year, divided by two), the retention plus the incoming and outgoing amounts, then multiplied by 100% [2]. Intention to stay is one of the significant determinants of turnover behavior. In Indonesia, hospitals are increasing, being mainly managed by private parties [3]. Most nursing personnel turnover was represented by women [4].

The current research aimed to explain the influence of cooperative behavior in the forms of perceived external prestige (PEP) and perceived internal respect (PIR) and how these were correlated with the intention to stay of nursing personnel. A high rate of turnover also causes uneven distribution of nursing personnel in every work unit. In certain work units, the shortage of nursing personnel is quite high compared to other work units. Besides the presence of several outgoing employees, the lack of workforce is also due to the insufficiency of the number of individuals recruited compared to the needs of the nursing personnel. According to Muchlis' research, organizational performance is a predictor of individuals and groups [5].

Since nurse turnover has become an issue in the healthcare system [6], the predictors must be identified. In this case, if the turnover is conducted involuntarily, then the organization's management in hiring the employees must be improved. This includes recruitment, selection, and training, as well as motivation improvement. This problem often occurs at the organizational management level, but prevention efforts have usually been carried out but have not been maximized [6, 7]. In addition, turnover can also be voluntary, which further becomes the main discussion in this research. The main reason for nurses' turnover is their voluntary employment status, not permanent employment, so there is no solid contract [8].

Carmeli defined organizational prestige as an organization's image [9]. In this case, an image is the opinion of someone concerning an organization. Someone can have judgments over an organization even though they do not have any interaction with it [10]. When an organization has a good image, it indicates that it has good prestige, maintaining its employees' commitment and satisfaction which can further lower their intentions of leaving the organization [9].

According to previous research [11], cooperative behavior can affect turnover behavior. In this case, cooperative behavior is defined as the individual behavior prediction resulting from group assessment. The theory of cooperative behavior divides group cooperation and individual behavior into two forms. The first refers to the individual involved in the group due to mandatory cooperative behavior, while the second refers to the individual involved in the group due to indirect demand from the rules/norms of the group (discretionary cooperative behavior). Tyler further explained that individual attitudes and values are mainly affected by this discretionary behavior. Therefore, an individual's identity in a group is essential because the stronger the individual's identity in the group, the easier the individual behaves according to the group's needs and requests. In addition, individual identity is also mainly affected by pride and respect [11].

Related to this, an individual can be identified through two assessments, the assessment of the group status (pride) and the assessment of status in the group (respect) [11]. This indicates that pride is defined as the individual's assessment regarding the status of the organization, while respect is defined as the assessment of the individual status in the organization. When the results of the assessment are positive, then it will increase group identification. This research further used the term perceived external prestige (PEP) to represent pride, as proposed in another study, as well as perceived internal respect (PIR) to represent PIR [12].

In previous research, job security was generally interpreted as the perception of financial guarantee in old age, whereas in this research, job security is interpreted as the perception of guarantee certainty regarding employee status in the hospital. The instrument was developed particularly on job security variables based on focus group discussions in the field. Some statements about why nursing personnel stays in the hospital were generally related to emotional factors (involving emotions) that refer to PEP and PIR and the job security of the hospital. The reasons for nursing personnel's preference to stay in the hospital were PIR, not yet passing the civil servant (job security), mutual respect for co-workers PIR, appreciation for each other's PIR, the fact that the hospital is one of the most well-known Islamic hospitals in Makassar PEP, working hard to pass the status of being a permanent employee (job security), be together with their colleagues PIR, financial problems (job satisfaction), and family atmosphere PIR like the nurses (job satisfaction). The management of nursing personnel was also adjusted to the needs and skill or skills of nurses (job satisfaction) and clarity of the recruitment system regarding the personnel in the hospital (job security). The organization's prestige in choosing the workplace and employee status becomes the basis of consideration to further examine PEP among nursing personnel, as well as PIR. Research studies are expected to address the high turnover rate in nursing personnel.

## MATERIAL AND METHODS

This quantitative research had a cross-sectional design, using a survey to study the causal relationship between the variables involved, and develop a model on the intention to stay of nursing personnel in the hospital. The study was carried out in five private and one government hospital, which was determined by the turnover rate trend of the hospitals in the last three years. Data collection was carried out for two weeks, from June until July. The population involved 214 non-permanent nursing personnel (freelancing, honorary and contractual) in five private hospitals. We conducted a survey (with a structured interview method) to patients/communities at each hospital to collect data on the level of PEP of the nursing personnel and people outside the hospital.

The inclusion criteria were nursing personnel working in the hospital and who served in all nursing units with non-permanent employment status (except internship), individuals who have worked in the hospital for at least one year, under 36 years old, and with no leave status or studying out of town. The sample included 214 nursing personnel, out of which 184 met the inclusion criteria, and the final sample consisted of 147 people.

To control the PEP of nursing personnel in the hospital, data were collected from the community/patient residing in the vicinity of the hospital location and from the patient at each study site. The inclusion criteria for the respondents of the survey were those who were at least 17 years old, with experience in visiting/using hospital services, with at least a junior high school/equivalent education (considered to have a fairly high education), and willing to participate in the survey. Meanwhile, the samples from the nursing personnel were collected based on proportionate random sampling, where the number of samples was collected based on the proportion of the number of nursing personnel in each hospital.

Based on the predetermined inclusion criteria and the results obtained at each hospital, the sample size varied at each hospital. This occurred because several individuals did not meet the criteria, worked no more than a year, were over 36 years old, were on leave status, resigned from nursing personnel, or were unwilling to participate in the research.

## RESULTS

Concerning the characteristics of the respondents, all respondents worked for 1 to 5 years in the hospital. This was also certainly related to the age of the respondents, who were relatively young. Most respondents worked in the nursing unit, and only a few of them worked in the intensive care unit (ICU), emergency room (ER), and operation room (OK) (Table 1). This is because some respondents refused to be interviewed on the unit, and the difficulty in setting up a special schedule for the interview because the time spent apart from work, was for rest and family.

**Table 1:** Socio-demographic characteristics of the participants.

Characteristics of nurses	Name of hospital										Total	
	RSIF		RSHM		RSIS		RSG		RSSM			
	n	%	n	%	n	%	n	%	n	%	n	%
**Gender**												
Male	2	7.7	4	15.4	3	4.6	0	0.0	4	14.3	13	7.6
Female	24	92.3	22	84.6	62	95.4	26	100.0	24	85.7	158	92.4
**Age group**												
21–25	16	61.5	5	19.2	14	21.5	14	53.8	18	64.3	67	39.2
26–30	9	34.7	12	46.2	46	70.8	12	46.2	10	35.7	89	52.0
31–35	1	3.8	9	34.6	5	7.7	0	0.0	0	0.0	15	8.8
**Marital status**												
Married	12	46.2	18	69.2	36	55.4	5	19.2	3	10.7	74	43.3
Not married	14	53.8	8	30.8	29	44.6	21	80.8	25	89.3	97	56.7
**Education level**												
SPK/SEDERAJAT	0	0.0	0	0.0	0	0.0	0	0.0	1	3.6	1	0.6
D3	16	61.5	21	80.8	42	64.6	25	96.2	18	64.3	122	71.3
D4/undergraduate of nursing/midwifery	10	38.5	5	19.2	23	35.4	1	3.8	9	32.1	48	28.1
**Employment status**												
Voluntary	0	0.0	2	7.7	4	6.2	0	0.0	0	0.0	6	3.6
Honorary	26	100	24	92.3	55	84.6	2	7.7	1	3.6	102	59.6
Permanent employee candidates	0	0.0	0	0.0	6	9.2	1	3.8	10	35.7	17	9.9
Contract	0	0.0	0	0	0	0.0	23	88.5	17	60.7	46	26.9
**Working period**												
1–5 years	24	92.3	15	57.7	40	61.5	21	80.8	26	92.9	126	73.7
5.1–10 years	2	7.7	8	30.8	24	36.9	5	19.2	2	7.1	41	24.0
>10 Years	0	0.0	3	11.5	1	1.6	0	0.0	0	0.0	4	2.3
**6. Working unit**												
UGD	1	3.8	1	3.8	8	12.3	0	0.0	4	14.3	14	8.2
ICU	0	0.0	1	3.8	0	0.0	0	0.0	3	10.7	4	2.3
OK	0	0.0	0	0.0	0	0.0	0	0.0	3	10.7	3	1.8
Nursing	25	96.2	24	92.4	57	87.7	26	100.0	18	64.3	150	87.7
**Ethnicity**												
Bugis	19	73.1	15	57.7	54	83.1	11	42.3	15	53.5	114	66.7
Makassar	7	26.9	11	42.3	7	10.8	6	23.1	1	3.6	32	18.7
Java	0	0.0	0	0.0	0	0.0	1	3.8	0	0.0	1	0.6
Manado	0	0.0	0	0.0	0	0.0	0	0.0	1	3.6	1	0.6
Mandar	0	0.0	0	0.0	3	4.6	0	0.0	0	0.0	3	1.8
Toraja	0	0.0	0	0.0	1	1.5	8	30.8	11	39.3	20	11.7

RSIF – Rumah sakit islam faisal; RSG – Rumah sakit grestelina; RSIS – Rumah sakit ibnu sina; RSHM – Rumah sakit haji makassar; RSSM – Rumah sakit stella maris.

The ethnicity distribution included Bugis, Makassar, Java, Mandar, Toraja, and Manado. Among these 6 ethnicities, Bugis ethnicity was mostly found in all hospitals, followed by Makassar.

As a control, surveys were conducted to patients and communities who used health services at the respective sites to determine their perceptions on the prestige of each hospital.

These are based on the final model results obtained from direct or indirect influence, presented in Table 2.

**Table 2:** The results of Confirmatory Factor Analysis (CFA).

Variable	Indicators	Validity	Reliability	
		^	AVE	CR
**Perceived External Prestige (PEP)**	1. High status; 2. The best; 3. The priority choice.	0.879 0.903 0.874	0.784	0.916
**Perceived Internal Respect (PIR)**	1. Be valued; 2. Care of well-being; 3. Contribution.	0.915 0.973 0.980	0.915	0.970
**Organizational Identification (OI)**	1. Self-categorization & labeling; 2. Value & goal sharing; 3. Belonging & membership.	0.937 0.985 0.921	0.899	0.964
**Intention to Stay (ITS)**	No construct test, only 1 indicator			
**Job Security (JS)**	No construct test, only 1 indicator			

**Figure 1:**
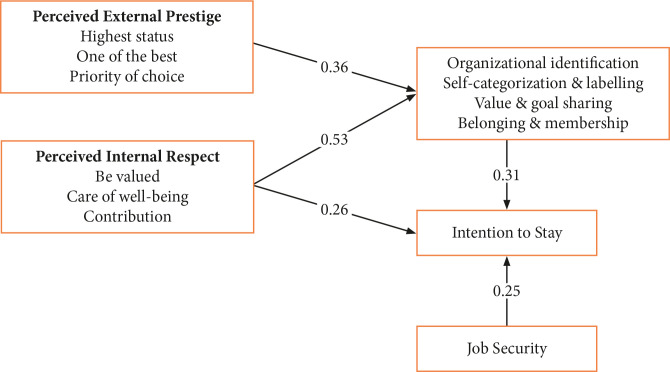
Intention to stay of nursing personnel model.

Figure 1 describes the final model of the intention to stay of nursing personnel in the hospital. The result from the goodness of fit was RMSEA=0.075 (value is accepted ≤0.08 (Browne dan Curdeck, 1993), GFI=0.928 (for standard ≥0.90), CFI=0.932 (for standard ≥0.90), CMN/DF=2.050 (for standard ≤2.00). This shows that the feasibility of the model was acceptable, which means there was a match between the model and the data (Table 2).

Based on the second model regarding the intention to stay of nursing personnel, perceived external prestige had a positive and significant effect on organization identification, with a path coefficient of 0.358 and probability significance (p) of <0.001. Perceived internal respect positively and significantly influenced organization identification, with a path coefficient of 0.527 and probability significance (p) of <0.001. The identification of the organization had a positive and significant impact on the intention to stay, with a path coefficient of 0.313 and probability significance (p) of 0.002. Perceived internal respect had a significant effect on the intention to stay, with a path coefficient of 0.319 and probability significance (p) of 0.261 (Table 3).

**Table 3:** Independent Variable to Dependent Variable.

Independent variable	Direct effect	Indirect effect	Total effect
Perceived External Prestige	-	0.358	0.358
Organization Identification	0.313	-	0.313
Perceived Internal Respect	0.261	0.527	0.789
Job Security	-	0.248	0.248

Increased perceived external presence (PEP) and perceived internal respect (PIR) could improve the organization identification (IO) of nursing personnel. The increased integration of perceived external prestige and perceived internal respect will improve the intention to stay nursing personnel. Increased job security will improve the intention to stay of nursing personnel. The developed model consisted of PEP, organizational identification, and PIR used to assess the intention to stay of nursing personnel which was fit as a model.

The cooperative behavior theory of a group in another study [11] explains that individuals identify with groups based on the evaluation of group status (pride) and based on their evaluations of their status in the group (respect). Positive identification results can motivate individuals to think positively about themselves and increase their commitment to the group (work unit). The group in the study mentioned above included several individuals with the same profession and social level. It is different from this research, where positive identification does not exist only in the work unit but also outside the group, strengthening the identification in the organization.

## DISCUSSION

Some of the results of this study are different compared to previous studies and differ from the hierarchical theory of needs [13]. In Maslow's Hierarchy of Needs pyramid, the safety and security aspects are at the second level, whereas self-esteem and self-actualization are at the fourth and fifth levels. Based on Maslow's theory, job security in this research can refer to safety and security, while value and contribution to the hospital can refer to self-esteem and self-actualization. However, the results of this study prove that valuable assets for the hospital and its contribution assessment are the main reasons nursing personnel can survive/stay in the hospital or through the needs of safety and security. Self-esteem and self-actualization are at the highest peak in Maslow's hierarchy of needs, so this theory does not apply to nursing power. It also proves that nursing personnel prioritizes fulfilling their self-esteem and self-actualization needs rather than the basic needs (psychological) despite nonpermanent employment status and the short duration of work (1–5 years).

Furthermore, based on the Job Demands-Resources model, the mediator between social support (job resource) and job satisfaction (organizational result) is work engagement. Meanwhile, recent studies assumed that social support is the moderator between work engagement and job satisfaction in nursing staff. This indicates that job satisfaction results from work engagement and social support from the supervisor and co-workers. Social support given by co-workers can improve work engagement, thus increasing the job satisfaction of nursing employees [14].

In addition to social support from the co-worker, emotional support from the supervisor also determines the employees' job satisfaction. However, no study has been conducted on assessing the effect of supervisor emotional support on job satisfaction. In this case, multilevel statistical techniques can help disentangle the effects of subjective assessments from those of group factors. Hence, nurses with high work engagement levels tend to have high job satisfaction levels, which strongly correlates with the supervisor's emotional support at the group level [15].

Furthermore, Terje Slåtten examined the role of organizational attractiveness (OA) from the perspective of hospital organizations. The examination revealed that the hospital manager's skill in developing an internal market-oriented culture was directly correlated to the hospital frontline employees since both have a direct effect on OA and an indirect effect on frontline employees' engagement, turnover intentions, and service-quality provision. Consequently, OA has a main role in affecting the frontline employees' perception regarding their opinion of whether their workplace is a great place to work. Therefore, it is important for the hospital manager to consider, focus, maintain, and cultivate OA as the hospital's main asset to make the hospital an attractive workplace in a very competitive market [16].

Additionally, the balance between personal life and professional life has become a discussion topic. This is due to the involvement of new technologies in personal life, the overlap between work time and family time, new organizational systems, and changes in the nature of work. This issue is further exacerbated by the intense working hours or schedules that are undoubtedly experienced by nursing professionals. Nurses are expected to work quickly and effectively so that they can meet the demand of public health needs. In addition, they also work in an industry with an intensive work environment, which operates nonstop for a whole day and a whole year and sometimes requires exceeding the standard 40 h a week of full-time employment [17].

The nursing profession in Europe will benefit significantly from this study's theoretical and practical outcomes. Theoretically, knowing how the Job Demands-Control-Support Model functions in a field like nursing, where complicated and dynamic activities are carried out, might significantly improve the work-life balance of professionals with demanding work schedules. The results of this study support the premise that work demands are not the only factors that affect work-life balance, particularly when employees feel that their employers are giving them job control and/or social support. As a result, the sector must examine the workplace elements that influence work-life balance. Human resource managers should look into new equipment to give employees control over their daily tasks, particularly in a profession like nursing where direct patient contact occurs and where the workers' ability to make decisions and exercise their freedom of action determines the quality of services provided. Greater autonomy will result from any advancement in this area (such as altering action protocols), which should improve the work-life balance (WLB). Managers should also encourage cooperation and foster a collaborative work environment built on a supportive company culture. By investing in the training of work teams, the organizational environment and social support will improve, leading to organizational changes (such as in the scheduling of work shifts) and employees' attitudes toward patient care. These techniques should lessen work-related stress and raise workers' WLB [17].

Downsizing would also affect middle managers, but they are required to demonstrate professionalism by performing their roles during the period of change. This research was conducted on companies experiencing downsizing in Indonesia. After downsizing, middle managers were satisfied with the process but did not show the expected work performance. This condition was different from a study by Bergström, who found that the survivors' organizational commitment increased after the downsizing process because the workers' program was well communicated [18].

The study by Purwaningrum showed the importance of developing a positive atmosphere during times of change. The positive atmosphere developed by the organization at the beginning of change did not affect the effective commitment to change sufficiently; therefore, a positive atmosphere must be sustainably grown. The organization had a major role in building a positive atmosphere by showing its commitment to change, and top management could be role models in change [19].

While there was a negative association between consensual organizational culture, sacrifice, appropriateness, and connection of job inclination, there was a positive correlation between turnover intention, resignation, and protective silence. The explanatory power of turnover intention was 47%, and the variables influencing it were current job experience, protective quiet, and sacrifice. Therefore, it will be required to create various programs to improve the communication pattern in the nursing organization and to give a diversified welfare system to reduce the intention of turnover [20].

Nurses' intention to remain in their current job can be influenced by authentic leadership and nursing organizational culture, particularly relation-focused culture. It is advised to employ a genuine leadership training program for nursing leaders and to make efforts to create a relation-focused culture at the hospital to retain nurses [21].

The long-term care of hospital nurses' level of happiness was not very high. Regarding the variables influencing their happiness, people reported feeling happier when their subjective health status was judged to be better, when they worked for an organization with a hierarchy-oriented culture, when they expressed a higher level of job satisfaction concerning autonomy, and when there were fewer task requests. Among these, autonomy-based work satisfaction had the most impact [21].

The findings of multiple studies revealed that the intention to stay is influenced by various circumstances (ITS). The most accurate indicators of an intention to quit (ITL) or stay were affective and normative commitment (ITS). ITS may be impacted by the interaction between emotive and cognitive ideas [22]. There are multiple reasons why nurses continue working in the hospital, including respect, prestige, job satisfaction, job environment, job opportunity, and job security [3].

## CONCLUSION

The importance of growing cooperative behavior through perceived internal respect and perceived external prestige can increase nurses' intention to stay. There is a need to develop a more in-depth study and a model regarding the intention to stay of nursing personnel in the ministry of health. The development of an intention-to-stay model is not limited to hospitals but to other types of health services, including different types of professions.

Furthermore, the hospital's management is expected to be more proactive in preventing a high turnover rate by adopting the intention to stay model, not only reactive and continuously recruiting, because it can lead to the inefficiency of human resource management and financial power in hospitals. In the formal form, the intention to stay model can be implemented and continued as part of the human resources management system in the hospital by involving the head of the team leader room and the nursing personnel themselves. In an informal manner, it can be done through daily interaction by discussing as an attempt to improve the intention to stay of nursing personnel.

## Data Availability

Further data is available from the corresponding author upon reasonable request.
